# Using Design Thinking Method in Academic Advising: A Case Study in a College of Pharmacy in Saudi Arabia

**DOI:** 10.3390/healthcare10010083

**Published:** 2022-01-02

**Authors:** Dalia Almaghaslah, Abdulrhman Alsayari

**Affiliations:** 1 Department of Clinical Pharmacy, King Khalid University, Abha 61441, Saudi Arabia; 2 Department of Pharmacognosy, King Khalid University, Abha 61441, Saudi Arabia; alsayari@kku.edu.sa

**Keywords:** design thinking, academic advising, peer academic advising, pharmacy students

## Abstract

Purpose: The current study was conducted to evaluate academic advising services in a pharmacy college in Saudi Arabia. This will result in developing solutions to overcome the identified challenges. Methods: Design thinking method uses five steps: empathising, defining, ideating, prototypes and testing. Results: Several issues were identified with students: limited awareness of academic rules and regulations; work-family life imbalance; lack of trust in academic advising and emotional support; unfamiliarity with different learning strategies; and lack of social life at the university. Discussion and conclusion: This study provides a model for enhancing students’ experiences with academic advising. It suggested several prototypes that have proven to be effective in enhancing students’ experiences in university life and how to overcome challenges. The prototypes include a peer academic advising club, personal development workshop and a series of lectures on college rules and regulations.

## 1. Introduction

Design thinking is a problem-solving approach that gained popularity in various fields [1]. Design thinking is defined as “a systematic approach that prioritises deep empathy for users’ desires, needs and challenges to fully understand a problem, with the aim of developing more comprehensive and effective solutions” [1]. It was also explained as a human-centred innovation process that emphasises observation, collaboration, fast learning, visualisation of ideas, rapid concept prototyping and concurrent business analysis [2,3,4]. Design thinking comprises five stages: empathising, defining, ideating, prototypes and testing [1].

The first step is empathy where deep non-judgmental understanding of the end-user’s needs, desires and values is established. This stage involves engaging, observation and empathising with humans to develop personal understanding of their experiences and motivations. Define: during this stage, identification of the core problem is performed by analyzing observations of the first stage. Ideation involves creating innovative solutions by developing alternative ways of looking at the core problems identified, usually conducted by a multi-disciplinary group. Prototype is an experimental phase that involves generating solutions to the identified issues before deciding the best alternative through an iterative process. The testing phase involves testing the alternative prototypes developed by obtaining feedback, and thus refining solutions [1].

Design thinking has been used more routinely in some fields such as engineering and architecture and more recently in education [5,6]. Literature indicated that academics have shown interest in embedding this approach in various educational settings [7]. Incorporation of the design thinking process in different educational environments resulted in specific advantages, including: implicating tactical experiences, enhancing empathy, limiting cognitive bias, encouraging playful leaning, enhancing motivation and concentration, creating resilience, producing creative and innovative solutions and fostering creative confidence [7].

Academic advising was defined as “the engagement of students to advance their educational experiences, and referring to the individual involved in this interaction with students as academic advisor” [8]. Student advising on curricular and extra-curricular opportunities has been recognised as an essential element in producing skillful, confident and critically-thinking students who are ready to join the job market [9]. Student faculty interaction was found to enhance student persistence, increase student retention rates and satisfaction with the college environment, thus contributing to their success [10]. However, student advising has been identified as a struggle in higher education worldwide. Students face challenges to connect within their curriculum, and often fail to connect outside their disciplines. Students are the key stakeholder in academic advising, but are often excluded from the solution aimed to overcome the issues they encounter [11].

Design thinking as a user-centered or human centered problem-solving approach was reported to have positive outcomes on academic advising by reaching an agreement on institutional dialogue, curriculum and cooperation by academics and students [9].

In this study, we report using design thinking framework to evaluate academic advising services in a pharmacy college in Saudi Arabia, better understand the challenges associated with academic advising and develop novel solutions to overcome the identified challenges.

## 2. Methodology

An empirical evaluation was conducted to understand the context of academic advising for undergraduate female pharmacy students using design thinking principles, adopted from Stanford University D-school [1]. A case study from the female campus of a pharmacy college in Saudi Arabia was used to identify the most important challenges.

The study was conducted between November 2019 and March 2020, using semi-structured interviews (preset of close-ended and open-ended questions) as in Table 1, which were conducted with the identified study sample. The interviews were carried out by a female researcher, who is an expert in qualitative studies and using the design thinking methodology. Participants were recruited through the purposive sampling technique. The inclusion criteria were as follows: a semester Grade Point Average (GPA) of ≤2.5 out of 5, and/or who have had warning as per the university regulations regarding minimum GPA (cumulative GPA is less than 2.00 but more than 1.00; or semester GPA is less than 2.00). GPA has been used in the literature to determine student academic performance [12]. Written informed consent was obtained from participants before the investigation was conducted. The identity of participants was kept confidential. A topic guide was developed and piloted with other students prior to the study. The interviews lasted about 20 min on average, and were conducted in the Arabic language before being translated into English. To ensure the translation was accurate, two scripts were translated back by another co-investigator who is a bilingual English-Arabic speaker. A member of study investigators (DA) preformed data analysis consistent with Braun and Clark’s approach [13]. The author (AA) provided insights and input during the analysis process. The initial stage involved (data-driven) inductive thematic analysis, where data was coded and classified, and similar categories were grouped into themes emerging from the data. Iterative analysis was employed, where data being analysed was constantly compared with earlier collected data. A total of five themes were developed. The Wilcoxon signed rank test was used to analyse the improvement in students’ GPA before and after the implementation of the prototypes [14].

**Figure 1:**
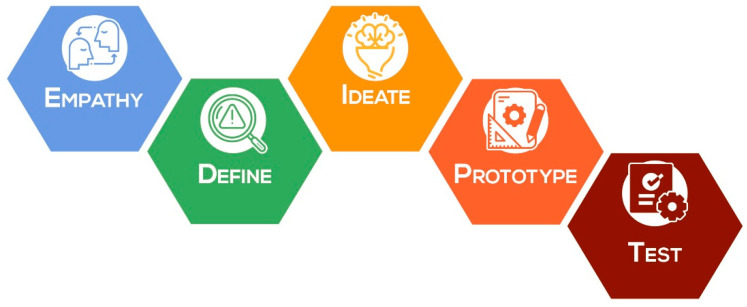
Stages of the design thinking process.

Ethical approval for this study was granted by the Ethical Committee of Scientific Research at King Khalid University (ECM#5824). The methodology is structured in five main stages, as illustrated in Figure 1.

### 2.1. Stage 1: Empathise

At the start of this case study, the study’s principal investigator (DA) interacted with students with low academic achievements. Based on students’ perspectives and pain points, a list of key issues was identified through the following steps:

**Observation:** investigator shadowed the students and observed their behavior during the classes of courses that they were enrolled in. This helped in identifying the struggles they faced with their academic studies.

**Engaging:** one-to-one interviews were conducted with students in the academic advising unit room in the college campus. To ensure that students were deeply engaged in the conversation, they were encouraged to take the lead and to elaborate more on their pain points in order to fulfill the exploration criterion of design thinking.

**Watching and listening**: observation and engaging were combined, for example, by asking students what they thought were the reasons for their struggles with academic life. Through many storytelling activities, the key problems with academic achievements identified strategies to improve their academic performance.

### 2.2. Stage 2: Define

After gathering information regarding the key challenges experienced by students, the final key issues were defined using the following two steps:

**Contextualise:** an analytical framework was developed using the themes that emerged from the interviews. A total of five themes were identified.

**Synthesise:** during this stage, the suggested solutions were structured. This was finalised by prioritising the key needs. The perspectives of the end users (students) were structured by combining three elements: user, needs and insight into an actionable problem stamen.

### 2.3. Stage 3: Ideate

The (define) stage created possible solutions to overcome the core issues identified through the following steps:

**Create:** expert panel expertise (academic advising unit members, high academic achieving students, teaching staff and a life coach) was gathered trough several brain-storming sessions. This allowed synergy of the panel to reach possible solutions. Promising ideas were developed by acknowledging limitations, accepting misunderstanding and including creative solutions.

**Prototype:** prototypes were created to facilitate the ideation phase. New ideas were created through several development stages. A few ideation methods were utilized, including brain writing, provocation, mind mapping and storyboards.

**Separate:** assessing ideas was not encouraged to facilitate creating more innovative and inspirational ideas.

### 2.4. Stage 4: Prototype

The gathered data directed the efforts to create possible solutions and techniques. The key issues and suggested solutions were determined, and then different prototypes were developed through the following steps:

**Build:** the model process was initiated, i.e., constructing something, coding preliminary solutions, as a useful beginning towards structuring a prototype.

**Variables:** each prototype was designed to overcome a certain problem when evaluated as to the way it improves students’ academic performance.

**Build from insights gained:** prototypes were developed and students’ behavior towards changes was monitored. Feedback was collected in the testing phase.

### 2.5. Stage 5: Testing (Validation with Collaborators)

The final stage involved testing the suggested solutions that addressed key issues and core needs.

**Observe and refine:** the proposed solutions were implemented to the end users (students), and their feedback was obtained.

**Create experience:** the new strategies were tested in terms of end user experience, rather than by their evaluation.

## 3. Results

### 3.1. Stage 1 Empathise and Stage 2 Define

**Figure 2:**
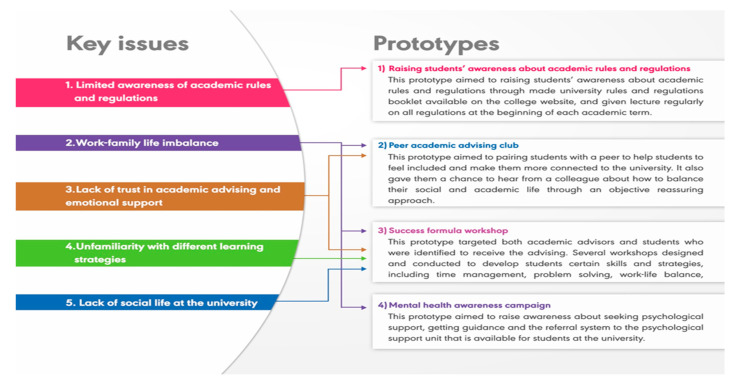
Identified key issues and suggested prototypes.

A total of 21 female pharmacy students were identified and recruited based on the inclusion criteria. All participants were between the ages of 22 and 26 years. One-to-one interviews were conducted by the principal investigator. Thematic analysis of the interview scripts was conducted independently by the study’s authors. Based on students’ perspectives and pain points, a list of core issues was identified and agreed on as follows (Figure 2):

1. Limited awareness of academic rules and regulations
2. Work-family life imbalance
3. Lack of trust in academic advising and emotional support
4. Unfamiliarity with different learning strategies
5. Lack of social life at the university

### 3.2. Stage 3 Ideate and Stage 4 Prototype

Six panel expert members participated in the design thinking workshop. Academic advising unit members (two females), high academic achieving students (two females), teaching staff (two males) and a female life coach. Participants created an empathy map as a group that explored feelings, needs, sayings, actions and sights of students who were eligible for academic advising. The notes were reviewed by the panel, and reached consensus on the features that made up the experience. Results were in line with the five identified core issues in the first two stages. The participants used brainstorming techniques to propose solutions that address the identified issues. A total of 20 ideas were discussed by the team. Major ideas aimed to help students find work-life balance, achieve more sense of belonging and be equipped with various learning strategies. The implemented solutions are summarised in Table 2 and Figure 2.

### 3.3. Raising Students’ Awareness about Academic Rules and Regulations

This prototype aimed to raise students’ awareness about academic rules and regulations through a university rules and regulations booklet available on the college website, and through lectures regularly given on all regulations at the beginning of each academic term.

### 3.4. Peer Academic Advising Club

This prototype was started by advertising positions for peer academic advisors. The inclusion criteria were as follows: GPA of ≥3.75, in level ≥ 4, had no ethical nor academic violation records, had good communication skills, had professional approach to the position, and were able to stay in the position for at least two academic semesters [15]. A total of 41 students met the inclusion criteria and were eligible for personal interview. Each interview was carried out by four academic staff members and aimed to assess their suitability for the rule as a peer academic advisor. Eighteen students were assigned for the job. The elected academic advisors were requested to have a profile that included their preferred mode of communication, number of students they are willing to be paired with, the student academic year (level) and the type of support they would be offering, as well as optional additional information about themselves. Their profiles were available on the college website. Students who needed support could choose their peer advisor by applying through a confidential electronic form that was available on the college web page. The academic advising unit would contact both students to be paired. The identified 21 students were successfully paired with academic advisors. 

### 3.5. Success Formula Workshop

This prototype targeted both academic advisors and students who were identified to receive the advising.

The workshop discussed the following topics/skills:

o Problem-solving techniques (fishbone, 5 why, brainstorming, check sheet, Pareto’s Principle of the 80/20 Rule, cost-benefit analysis).
o Work-life balance (work-life triangle, reasons for work-life imbalance, how working hours have changed over time, signs to watch, consequences of losing your work-life balance, work-life balance tips and techniques, time management skills, the Eisenhower matrix, procrastination).
o Real-life examples of students who succeeded in work-life balance (married with kids).
o Real-life examples of students involved in volunteer work.
o Personal values and decision-making.
o Communication skills and volunteer work.
o Learning how to learn (learning in high school versus learning in college, learning taxonomies, strategies for test-taking).

### 3.6. Mental Health Awareness Campaigns

Two mental health literacy campaigns, “We are with you” and “Never be ashamed (La tathreeb)”, were carried out in the college campus and aimed to raise awareness about seeking psychological support and getting guidance and the referral system to the psychological support unit that is available for students at the university.

#### Testing

The number of students who used the services was used as an indicator of the success. Twenty-one students who were initially identified were successfully paired with peer academic advisors. A survey was conducted to assess the success formula workshop (Table 3 and Table 4). Students’ GPA were used as a measure for assessing the success of the prototypes. The Wilcoxon signed rank test shows a significant difference between students’ GPA before and after the implementation of the prototypes (z = −3.8; *p* = 0.0001), as shown in Table 5, indicating improvement on students’ overall performance. In addition, seven students have graduated from the program, which shows a significant difference between students’ GPA before and after the implementation of the prototypes (z = −3.8; *p* = 0.0001).

## 4. Discussion

The current study was conducted to evaluate students’ experiences with academic advising in a pharmacy college in Saudi Arabia using design thinking principles. The design thinking framework was chosen to provide a deep understanding of students’ feelings, values and pain points. This human-centered approach puts the end-user’s desires and needs as priority in developing solutions. In this scenario, students have low academic achievements and are struggling with their studies, and in some cases have family issues caused by a lack of work-life balance, psychological issues related to perceived social isolation, a lack of sense of belonging and a lack of trust in people around them, causing them to withdraw and hold back.

A recent study conducted in a pharmacy college in Saudi Arabia indicated that mental health problems such as stress, depression and anxiety could lead to a long-term negative impact on physical social and mental health, as well as on academic preformance. An interesting finding was reported, that pharmacy students were likely to seek help from professional health personnel i.e., a psychologist or a psychiatrist, or from a friend, but they would not seek help from teaching staff [16]. Another study which investigated help seeking behavior among Saudi dental students reported that the majority relied on their colleagues’ advice regarding academic issues [17]. Similar findings were reported in this investigation, which was the main drive behind establishing the peer academic advising club, where the role of teaching staff in providing support and help is limited because students are often hesitant, embarrassed or even reluctant to admit they need help from academics [12].

This prototype focused mainly on overcoming the identified issues, i.e., lack of social life at the university, work-life imbalance and lack of trust in academic members. Pairing students with a peer helped students to feel included and made them more connected to the university. It also gave them a chance to hear from a colleague about how to balance their social life and academic life through an objective reassuring approach. Previous studies have shown that peer academic advising equips students with employment skills, such as confidence, self-esteem and altruistic values [12,13]. On the other hand, peer advisors also benefit from their role by gaining customer-services experience, advancing their interpersonal communication, leadership and advising skills [18].

Another prototype was the success formula workshop, which also targeted the same problems by having a trusted professional talking to students about developing certain skills and strategies, including time management, problem solving, work-life balance, avoiding procrastination, decision-making based on personal values, volunteer work, interpersonal communication and learning strategies; both peer advisors and advisees were very satisfied with workshop.

Having a multidisciplinary team generating ideas and constructing prototypes helped in finding solutions to the other identified issues, such as limited awareness of the academic rules and regulations and unfamiliarity with different learning strategies. These two issues required the participation of teaching staff as the main stakeholders.

The traditional role of academic advisors involves helping students in choosing courses, support decisions that comply with campus policies and procedures with the ultimate goal of students completing degrees [19,20,21]. These basic elements of advising were assured by a prototype that involved giving lectures about the college rules and regulations, mainly about selecting courses, dropping and adding courses, minimum credit hours, minimum GPA and other policies that students identified as priority to their success. Communication skills was found to be the most important competency for entry-level advising [20]. Thus, selecting the academic advisor responsible for providing these lectures was solely based on their social skills, as it has been reported that other elements, including knowledge of curriculum, technology, teamwork and critical thinking, were less important [20].

This study provides a model for enhancing students’ experiences with academic advising. It suggests several prototypes that have proven to be effective in enhancing students’ experiences in university life and overcoming challenges; work-life imbalance, lack of social life at the university, limited awareness of the academic rules and lack of trust in academic members. The study’s findings and prototypes could be generalisable to enhance students’ experiences in similar settings, i.e., national colleges and universities, which are facing the same identified issues with academic advising.

Studies have revealed that using the design thinking approach is associated with several disadvantages, including a lack of experience in using this approach, which might result in a lack of mastery and creative confidence, produce shallow ideas, lead to mistakes in prioritisation and also frustration and anxiety when first introduced, as well as creative overconfidence and conflicts among teams, an inability to move from idea generation to development of a final product, and ideas overproduction over-evaluation [7].

In the same way, some limitations have been identified, such as the sustainability of the peer academic advising club not being assessed, as peer academic advisors were not paid to perform the role and other confounding variables might have affected students GPA, and having a control group would have prevented students from benefiting from the newly implemented services.

## 5. Conclusions

This research developed a model for academic advising system at a pharmacy college in Saudi Arabia using a design thinking framework. This model consists of a few prototypes that have been successfully implemented. It incorporates a peer academic advising club, personal development workshop and a series of lectures on college rules and regulations.

## Figures and Tables

**Table 1:** Interview guide.

Close-Ended Question	
Name	
University ID	
College	
Level	
Age	
Marital status	
Home address	
Health status	o Good o Has a medical condition
Living condition	o With family o On their own
**Open-Ended Question**	
Why in your opinion did you obtain this GPA?	
Do you have any particular issue that has been affecting your academic achievements? Would you be comfortable to share it with me?	
How do you think we can help you (the college, the academic advising unit, academic staff)?	

**Table 2:** Academic advising framework.

End User Perspectives	Key Problem	Key Management
**Student 3**: I did not know much about registration in terms of how many courses [credit hours] I’m supposed to have. **Student 11**: I wanted to withdraw from a course, but my credit hours would’ve been fewer than 12 and that wasn’t allowed, but I didn’t know that at that time. **Student 7**: My GPA was less than 2.5 and I got a warning. I wasn’t aware that I’d get one.	**Limited awareness of the academic rules and regulations**	**Raising students’ awareness about academic rules and regulations.**
**Student 5**: I live far from campus—about 1.5 h—and having a baby made things even more difficult. Finding a balance between family and studies was challenging. **Student 8**: I’m expected to help my family with the house chores and sometimes I find it really stressful to manage, especially during mid-terms. **Student 19**: As a young married woman, I’m struggling to find a balance between university life and family life. **Student 20**: Being married while studying is hard, but I don’t want to go into details.	**Work-family life imbalance.**	**Success formula workshop** **Peer academic advising club** **Mental health awareness campaign**
**Student 4**: Sometimes they act like they care, but when I have conflicts in the midterm exam, they don’t seem to care. **Student 13**: I don’t feel comfortable talking freely with them [academics]. **Student 15**: I don’t think I can trust them [academics].	**Lack of trust in academic advising and emotional support.**	**Success formula workshop** **Peer academic advising club**
**Student 2**: I don’t know how to study; I spend so much time in studying. **Student 6**: All the time I spend studying doesn’t seem to make any difference. This is my last chance; I got my last warning to be expelled from university.	**Unfamiliarity with different learning strategies.**	**Success formula workshop** (Learning how to learn).
**Student 9**: I’m between level 5 and level 6. I don’t know anyone in the class. **Student 11**: I feel isolated, because I’m a repeater and all my batch had already graduated. **Student 15**: I don’t feel I belong, I’m out of place. I don’t have any friends who would support me.	**Lack of social life at the university.**	**Success formula workshop.**

**Table 3:** Quantitative evaluation of participants’ views of the workshop N = 27.

Statement	Strongly Agree *n* (%)	Agree *n* (%)	Neutral *n* (%)	Disagree *n* (%)	Strongly Disagree *n* (%)
The workshop was applicable to my job	18 (69.2)	7 (26.9)	1 (3.8)	0	0
I will recommend this workshop to others	22 (81.5)	4 (14.8)	0	1 (3.7)	0
The programme was well placed within the allotted time	15 (55.6)	6 (22.2)	4 (14.8)	2 (7.4)	0
The instructor was a good communicator	22 (81.5)	3 (11.1)	1 (3.7)	1 (3.7)	0
The material was presented in an organised manner	21 (77.8)	5 (18.5)	0	0	1 (3.7)
The instructor was knowledgeable on the topic	17 (65.4)	6 (23.0)	2 (7.7)	0	1 (3.8)
I would be interested in attending a follow-up, more advanced workshop on this subject	22 (88)	3 (12)	0	0	0
Overall programme	18 (72)	7 (28)	0	0	0

**Table 4:** Participants’ overall impressions of the workshop.

Attendes Opinions about Success Formula Workshop
▪ Wonderful workshop, I didn’t feel the time ▪ A new view on academic life ▪ I enjoyed the talks about volunteering ▪ Everything was perfect ▪ The speakers were inspirational ▪ Everything was nice, I hope you give such workshops ▪ The presentations were great and beneficial ▪ The speakers were fun and courteous ▪ The atmosphere, the team spirit and the speakers were perfect ▪ Great, knowledgeable instructors. More detailed oriented in future workshops ▪ It was great overall ▪ The entire programme was extremely useful; time flew and I learned a lot. ▪ The topics were excellent, the speakers were well-informed and had valuable knowledge. ▪ Sharing experiences was inspirational, especially participants’ stories about volunteer work and work-life balance

**Table 5:** Wilcoxon signed rank test results of students’ GPA pre- and post-implementation of the prototypes.

Post-Implementation—Pre-Implementation	N *	Mean Rank	Sum Rank	z	*p* Value
Negative ranks	1	1	1	−3.8	0.0001
Positive ranks	18	10.5	189		
Equal	0	-	-		
Total	19	-	-		

* Two students have withdrawn from the program.

## Data Availability

The data presented in this study are available on request from the corresponding author.
